# Whose alliance shapes whose appraisal? Longitudinal actor–partner associations between therapeutic alliance and session quality, clinical severity, and global improvement among Filipino client–therapist dyads

**DOI:** 10.1080/28324765.2026.2716621

**Published:** 2026-08-11

**Authors:** John Robert C. Rilveria

**Affiliations:** a Department of Psychology, University of the Philippines, Quezon City, Philippines

**Keywords:** Actor–partner interdependence model, Filipino client–therapist dyads, psychotherapy, session quality, therapeutic alliance

## Abstract

This study examined longitudinal actor–partner associations between client- and therapist-rated alliance and three appraisal domains: session quality, clinical severity, and global improvement. Using an intensive longitudinal dyadic design, 54 unique Filipino client–therapist dyads were followed across eight to ten psychotherapy sessions. Longitudinal actor–partner interdependence models tested whether each dyad member’s alliance rating at session t predicted their own and the other member’s appraisal at session t + 1, controlling for prior appraisal, observed session number, and between-dyad alliance means. Descriptive trajectories showed that alliance ratings were relatively stable, session quality fluctuated across sessions, clinical severity declined over time, and global improvement increased across observed treatment sessions. Actor effects were supported primarily for session quality: stronger-than-usual client-rated alliance predicted higher subsequent client-rated session quality, and stronger-than-usual therapist-rated alliance predicted higher subsequent therapist-rated session quality. Therapist-rated alliance also predicted lower subsequent therapist-rated clinical severity. Partner effects were not statistically significant across the primary models. Cross-domain comparisons indicated that actor effects were most evident for session quality, whereas partner effects did not vary meaningfully across appraisal domains. Findings suggest that alliance fluctuations are most consistently associated with each dyad member’s own subsequent session appraisal and highlight the value of bidirectional alliance monitoring in psychotherapy practice.

## Introduction

The therapeutic alliance remains one of the most robust relational correlates of psychotherapy outcome. In Bordin's (1979) pantheoretical formulation, alliance comprises agreement on therapeutic goals, agreement on therapeutic tasks, and the emotional bond between client and therapist. Meta-analytic and systematic reviews show that stronger alliances are associated with more favourable outcomes across treatment orientations, populations, and delivery formats (Flückiger et al., 2018; Saxler et al., 2024). Yet alliance is not a property of the client or therapist alone. It is co-constructed within a relationship in which the two participants occupy different roles and may interpret the same therapeutic interaction differently. Thus, the question is not only whether alliance predicts outcome, but whose alliance perception matters, for whose appraisal, and under what relational conditions.

The actor–partner interdependence model (APIM; Cook & Kenny, 2005) provides a framework for examining these distinguishable but interdependent perspectives. An actor effect refers to a person’s predictor score being associated with that person’s own outcome, whereas a partner effect refers to that person’s predictor score being associated with the other dyad member’s outcome. In psychotherapy, these paths help distinguish whether a client’s or therapist’s alliance perception primarily informs their own subsequent appraisal, crosses over to the other member’s appraisal, or does both. Importantly, APIM paths are predictive associations and should not, by themselves, be interpreted as causal influence. This framework aligns with counselling psychology’s emphasis on the therapeutic relationship as a central and cross-theoretical mechanism of change (Gelso, 2014) and with evidence-based relationship research that conceptualises therapeutic change as reciprocal, interpersonal, and perspectival rather than purely individual (Norcross & Wampold, 2018).

Psychotherapy APIM research has produced a differentiated rather than uniform account of actor and partner processes. One recurring pattern is the relative prominence of actor effects and the perspective-specific nature of therapeutic appraisals. Client and therapist perceptions of the alliance or broader therapeutic relationship often predict their own subsequent evaluations more consistently than those of the other dyad member. For example, Kivlighan et al. (2014) found actor associations between client and therapist alliance ratings and their respective session evaluations, although selected partner effects varied according to client reliable change. Similarly, Markin et al. (2014) found that clients’ subsequent session-quality ratings were predicted primarily by their own prior perceptions of the real relationship, whereas therapists’ evaluations reflected both their own and their clients’ prior relational appraisals. Zilcha-Mano et al. (2016) likewise showed that within-treatment increases in patient-rated alliance predicted both patient- and therapist-rated session outcomes, whereas therapist-rated alliance changes were more consistently associated with therapists’ own session outcomes. Together, these findings suggest that actor and partner effects may differ by dyad member, appraisal domain, and treatment progress, with client and therapist perspectives providing related but noninterchangeable information.

A second theme concerns the temporal stability, variability, and co-development of alliance perceptions. Longitudinal studies consistently indicate that both client and therapist alliance ratings show meaningful session-to-session continuity, but they also reveal circumstances under which one person’s alliance experience may shape the other’s subsequent perception. Lin et al. (2024), using dynamic structural equation modelling of 10,720 session-level observations from 236 clients and 45 therapists, found significant actor effects for both client- and therapist-rated alliance, indicating substantial temporal stability. They also identified a client-to-therapist partner effect: stronger-than-usual client alliance during one session predicted higher therapist-rated alliance at the next session. Lower variability in both perspectives was associated with better client functioning, while stronger therapist actor and partner dynamics were related to improvement. Escudero et al. (2022) similarly found that alliance ratings increased and became more convergent across 12 sessions with maltreated adolescents, with therapists appearing responsive to adolescents’ preceding alliance experiences, particularly in more successful cases. Li (2022) also identified patterns of temporal self-consistency and mutual influence in client- and therapist-rated alliance, with greater therapist consistency and a subgroup characterised by reciprocal influence that was associated with greater symptom improvement. These findings suggest that partner responsiveness may emerge most clearly when alliance itself is modelled as a dynamically co-developing process and may depend on population, treatment structure, clinical success, or the particular temporal window examined.

A third theme is the importance of distinguishing stable between-dyad differences from session-specific within-dyad fluctuations. Dyads who generally report stronger alliance, more favourable expectations, or greater relational consistency may differ from other dyads, but these between-dyad differences do not necessarily imply that momentary changes in one member’s appraisal predict changes in the other member’s subsequent experience. Constantino et al. (2020) identified longitudinal actor–partner associations between outcome expectations and alliance, together with indirect associations with treatment outcome. In contrast, Gaines et al. (2025), using repeated client and therapist ratings across as many as 16 weeks of naturalistic psychotherapy, found no significant within-dyad actor or partner effects from outcome expectations to alliance. At the between-dyad level, however, clients’ and therapists’ average outcome expectations predicted their own average alliance ratings but not their partners’ ratings. This distinction underscores the need to separate enduring dyadic characteristics from session-specific deviations when interpreting actor and partner associations.

Collectively, the literature indicates that dyadic effects vary according to whose perspective is assessed, whether alliance is treated as a predictor or outcome, the temporal level of analysis, the stage and structure of treatment, the clinical population, and the outcome domain. Existing studies have primarily examined alliance co-development, relational predictors of session evaluation, or reciprocal associations between alliance and outcome expectations. The present study extends this work by separating within-dyad alliance fluctuations from between-dyad alliance means and testing whether each member’s alliance perception predicts both their own and their partner’s subsequent appraisals across three distinct domains: session quality, clinical severity, and global improvement. It therefore examines not only whether client and therapist alliance perspectives influence one another over time, but whose alliance perception predicts whose later appraisal of the session and the course of clinical change.

## Gaps in the current literature and contributions of the present study

The present study addresses several gaps in the existing literature. First, less is known about how client and therapist ratings of their alliance prospectively relate to multiple, conceptually distinct appraisals of psychotherapy. Session quality captures the immediate experiential value of a session, clinical severity reflects current psychological difficulty and impairment, and global improvement represents perceived change over time. These domains are complementary but noninterchangeable: a session may be experienced as helpful despite persistent severity, and clients and therapists may differ in their judgements of progress. The present study therefore examines alliance in relation to all three domains rather than relying on a single indicator of therapeutic process or change. Second, the study distinguishes session-specific alliance fluctuations from stable between-dyad differences. Longitudinal APIM permits repeated observations to be modelled while preserving the distinguishable roles of client and therapist (Gistelinck & Loeys, 2019; Lafit et al., 2022). This makes it possible to test whether stronger- or weaker-than-usual alliance ratings predict subsequent appraisals beyond each dyad member’s typical alliance level and prior appraisal. Such separation is important because alliance and related interpersonal processes vary across treatment and may carry different implications at the within- and between-dyad levels (Iovoli et al., 2025; Li, 2022). Third, the study focuses on Filipino client–therapist dyads, who remain underrepresented in longitudinal dyadic alliance research. Alliance perceptions are culturally mediated (Davis et al., 2018), and Filipino psychotherapy involves culturally situated expectations concerning shared realities, trust, authority, reciprocity, disclosure, and relational harmony (Rilveria, 2024). Examining client and therapist perspectives separately is therefore necessary for identifying divergences that may otherwise be obscured by a single aggregate alliance score. Finally, the study has implications for routine alliance monitoring and feedback-informed practice. Clinical feedback can support progress tracking, early identification of deterioration or disengagement, and responsive treatment decisions (McAleavey et al., 2024). Because client and therapist alliance perspectives may carry different implications for session evaluation and outcome (Kivlighan et al., 2014; Zilcha-Mano et al., 2016), alliance scores should not be treated merely as satisfaction ratings or therapist performance indicators. Instead, discrepant or asymmetric appraisals may serve as relational signals for supervision, collaborative enquiry, rupture repair, and culturally responsive practice (Boswell et al., 2025; Orlowski et al., 2025). In this way, the study extends relationship-centred and evidence-based responsiveness models by examining whose alliance perception predicts whose subsequent appraisal across distinct domains of psychotherapy process and change (Norcross & Wampold, 2018; Stefana et al., 2024).

## Research objectives

The general objective of the present study is to examine longitudinal actor–partner associations between client- and therapist-rated therapeutic alliance and their appraisals of session quality, clinical severity, and global improvement among Filipino client–therapist dyads. Specifically, the study aims to: (1) describe longitudinal patterns in client- and therapist-rated therapeutic alliance, session quality, clinical severity, and global improvement across observed psychotherapy sessions; (2) test client and therapist actor effects by examining whether each dyad member’s alliance rating at a given session predicts their own appraisal of session quality, clinical severity, and global improvement at the following session; (3) test client-to-therapist and therapist-to-client partner effects by examining whether clients’ alliance ratings at a given session predict therapists’ subsequent appraisals of these domains and whether therapists’ alliance ratings predict clients’ subsequent appraisals; (4) evaluate within-dyad alliance fluctuations by determining whether session-specific increases or decreases in client- and therapist-rated alliance predict subsequent appraisals after controlling for prior appraisal scores, observed session number, and between-dyad alliance means; (5) compare actor and partner associations across appraisal domains by testing whether the magnitude and direction of alliance effects differ among session quality, clinical severity, and global improvement; and (6) determine whether actor effects are stronger than partner effects within each appraisal domain.

## Methodology

### Research design

The study employed an intensive longitudinal dyadic design involving 54 unique Filipino client–therapist dyads observed across eight to ten psychotherapy or counselling sessions. The dyads were distinguishable, with each dyad composed of one client and one therapist occupying theoretically distinct roles within the therapeutic relationship. Each therapist contributed only one client to the study; thus, the analytic structure involved repeated session-level observations nested within independent client–therapist dyads, without an additional therapist-level nesting structure. The study used the longitudinal actor–partner interdependence model (L-APIM) to examine whether each dyad member’s therapeutic alliance rating was associated with their own subsequent appraisals and the other dyad member’s subsequent appraisals across observed sessions. It is important to note that the ‘observed session number’ referred to the ordinal position of a session within the study assessment window (1–10), not its absolute position in the client’s full course of psychotherapy. Because dyads enrolled while already engaged in treatment, the first observed study session could occur at different stages of therapy across dyads. Absolute course-of-treatment session number at enrolment and the number of sessions completed before enrolment were not collected.

### Research methods

In collecting the data, the study used repeated post-session survey methods. Clients and therapists independently completed parallel measures immediately after each observed session. The repeated measurement occasions allowed the study to examine both stable between-dyad differences and within-dyad session-to-session fluctuations in therapeutic alliance and appraisal outcomes. Therapeutic alliance served as the primary dyadic predictor. The outcome domains included session quality, clinical severity, and global improvement. Session quality was treated as the primary outcome domain because it is most proximal to the session-level alliance process. Clinical severity and global improvement were treated as secondary clinical appraisal domains.

### Research participants

The analytic sample consisted of 54 unique client–therapist dyads, comprising 54 clients and 54 therapists. Each client was paired with a different therapist, resulting in 108 individual participants. Dyads were recruited from psychotherapy or counselling settings in Metro Manila, Philippines, including private practice, university-based clinics, community clinics, and other outpatient mental health service settings.

Clients were eligible to participate if they met the following criteria: (a) they were at least 18 years old; (b) they identified as Filipino and were receiving psychotherapy or counselling within a Filipino clinical context at the time of the study; (c) they were currently engaged in individual psychotherapy or counselling with a participating therapist; (d) they were expected to continue treatment for at least eight sessions after enrolment; and (e) they were willing to complete measures across eight to ten consecutive sessions. Clients were excluded if participation was judged by the therapist or research team to substantially interfere with ongoing treatment, intensify acute distress, or compromise clinical safety. Clients receiving crisis-only, assessment-only, group, couple, or family intervention were excluded because the dyadic model requires an ongoing individual client–therapist relationship. Therapists, in turn, were eligible to participate if they met the following criteria: (a) they were licensed mental health professionals providing psychotherapy or counselling services; (b) they were currently conducting individual psychotherapy or counselling with an eligible client at the time of the study; (c) they agreed to complete post-session questionnaires independently after each observed session; and (d) they were willing to participate with only one client–therapist dyad in the study to preserve dyadic independence. Participating therapists represented different theoretical orientations, including cognitive-behavioural, psychodynamic, humanistic–existential, integrative, family systems-informed, narrative, or other counselling and psychotherapy approaches. Treatment orientation, years of clinical experience, treatment modality, and usual session frequency were recorded for descriptive purposes.

#### Descriptive profile of participants

The analytic sample included 54 Filipino client–therapist dyads. The mean number of observed sessions was 8.72 (SD = 1.09) across the dyad sample. The mean number of complete paired session assessments per dyad was 7.19 (SD = 1.24). Complete lagged transitions were available for the session-quality (*n* = 208), severity (*n* = 283), and improvement (*n* = 236) analyses.

Clients were, on average, 27.67 years old (SD = 7.08, range = 18–44). Most identified as female (63.0%), followed by male (31.5%) and nonbinary or another gender identity (5.6%). The most common presenting concerns were anxiety or stress (31.5%), academic or work stress (20.4%), interpersonal conflict (16.7%), depressive symptoms (14.8%), adjustment or life transition concerns (9.3%), and trauma-related concerns (7.4%). Slightly more than half of clients had prior psychotherapy experience (53.7%), while 40.7% reported no prior psychotherapy experience and 5.6% had unknown prior treatment history. A little over one-third of clients were currently taking psychiatric medication (37.0%).

Therapists were, on average, 36.26 years old (SD = 6.16, range = 25–49). Most identified as female (64.8%), followed by male (29.6%) and nonbinary or another gender identity (5.6%). Therapists had an average of 7.39 years of clinical experience (SD = 3.58, range = 1.40–17.40) and reported diverse theoretical orientations, including psychodynamic/integrative (27.8%), schema-informed (25.9%), CBT/integrative (18.5%), humanistic/person-centred (18.5%), and eclectic/integrative (9.3%) approaches. Nearly half of dyads were seen in university clinic settings (48.1%), followed by community mental health centres (20.4%), hospital outpatient services (16.7%), and private training clinics (14.8%). Treatment was delivered mostly online for 44.4% of dyads, hybrid for 27.8%, and mostly in person for 27.8%.

### Research instruments

#### Demographic and treatment information form

Clients completed a brief form assessing age, gender, educational background, employment status, presenting concern, previous counselling or psychotherapy experience, and current psychiatric medication or other mental health service use. Therapists reported age, gender, profession, years of clinical experience, theoretical orientation, treatment setting, modality, and observed session number. For each assessment occasion, the ordinal study-observation number and session modality were recorded. Absolute course-of-treatment session number and the number of weeks already spent in treatment were not available.

The complete alliance measures (client and therapist versions of the WAI-SR) and appraisal measures (SES-P-SF and SES-T-SF, PGI-S and CGI-S, and PGI-I and CGI-I) are provided in the Supplementary Material.

#### Working alliance inventory–short revised

Therapeutic alliance was assessed using the parallel client and therapist versions of the 12-item Working Alliance Inventory–Short Revised (WAI-SR; Hatcher & Gillaspy, 2006). Each form contains four items assessing agreement on therapeutic goals, four assessing agreement on therapeutic tasks, and four assessing the emotional bond. Sample client items include ‘___ and I collaborate on setting goals for my therapy’ (Goals), ‘What I am doing in therapy gives me new ways of looking at my problem’ (Tasks), and ‘I feel that ___ appreciates me’ (Bond). Corresponding therapist items include ‘___ and I have collaborated on setting goals for these sessions,’ ‘___ and I both feel confident about the usefulness of our current activity in therapy,’ and ‘I appreciate ___ as a person.’ All items were rated on a 5-point frequency scale: 1 = seldom, 2 = sometimes, 3 = fairly often, 4 = very often, and 5 = always. Possible mean scores ranged from 1 to 5, with higher scores indicating stronger perceived agreement on goals and tasks and a stronger therapeutic bond. In the present data, internal consistency was *α* = .97 for the client form and *α* = .95 for the therapist form.

#### Session evaluation scale—patient and therapist short forms

Session quality was assessed using the parallel three-item ultra-short forms of the Session Evaluation Scale for patients and therapists (SES-*P*-SF and SES-T-SF; Hill & Kellems, 2002; Stefana & Hill, 2025). The client form included the items ‘I thought that this session was helpful,’ ‘I did not think that this session was valuable,’ and ‘Rate the overall effectiveness of this session.’ The corresponding therapist items were ‘I thought that this session was helpful for my patient,’ ‘I did not think that this session was valuable for my patient,’ and ‘Rate the overall effectiveness of this session for my patient.’ For the first two items, responses were provided on a 5-point Likert scale: 1 = strongly disagree, 2 = disagree, 3 = neutral, 4 = agree, and 5 = strongly agree. The overall-effectiveness item was rated from 1 = not effective to 5 = effective, with higher intermediate values reflecting progressively greater perceived effectiveness. The negatively worded value item was reverse-scored before scale computation. The mean of the three items represented session quality, yielding possible scores from 1 to 5, with higher scores indicating a more favourable appraisal of the session. Clients and therapists independently completed their respective forms immediately after each observed session. Internal consistency was *α* = .93 for both the client and therapist forms.

#### Global impression of severity

Clients completed the Patient Global Impression–Severity (PGI-S) item that asked, ‘Please choose the response below that best describes the severity of your overall psychological or emotional condition after your treatment session.’ Responses were rated on a 7-point scale designed to parallel the clinician-rated severity measure: 1 = no current psychological or emotional difficulty, 2 = borderline or minimal difficulty, 3 = mild difficulty, 4 = moderate difficulty, 5 = marked difficulty, 6 = severe difficulty, and 7 = extremely severe difficulty. Therapists completed the Clinical Global Impression–Severity scale (CGI-S; Busner & Targum, 2007), which asks, ‘Considering your total clinical experience with this particular population, how mentally ill is the patient at this time?’ The response options were 0 = not assessed, 1 = normal, not at all ill, 2 = borderline mentally ill, 3 = mildly ill, 4 = moderately ill, 5 = markedly ill, 6 = severely ill, and 7 = among the most extremely ill patients. Ratings of 0 indicated that severity was not assessed and were treated as missing rather than as substantive severity scores. Accordingly, analysed scores for both client- and therapist-rated severity ranged from 1 to 7, with higher values indicating greater global psychological or clinical severity. Because these were single-item measures, internal consistency was not applicable. Prior work supports the clinical utility of the CGI-S (Busner & Targum, 2007) and the construct validity and responsiveness of the PGI-S in specific clinical populations (Viktrup et al., 2012).

#### Global impression of improvement

Beginning with the second observed session, clients completed the Patient Global Impression–Improvement scale and therapists completed the Clinical Global Impression–Improvement scale (PGI-I, CGI-I; Busner & Targum, 2007). These measures were adapted so that the reference point was the preceding session rather than the baseline. The client item asked, ‘Compared to the previous session, how would you describe how much you have improved?’ The therapist item instructed clinicians to ‘Rate total improvement whether or not, in your judgement, it is due entirely to the treatment’ and asked, ‘Compared with the client’s condition during the previous session, how much has the client changed?’ The original response scale for both measures was 1 = very much improved, 2 = much improved, 3 = a little improved or minimally improved, 4 = no change, 5 = a little worse or minimally worse, 6 = much worse, and 7 = very much worse. A response of 0 = not assessed was also available on the clinician form and was treated as missing. Thus, the original substantive response range was 1 to 7, with lower scores indicating greater global improvement and higher scores indicating deterioration. For analysis, global improvement ratings were reverse-coded. The resulting analytic scale ranged from 1 to 7: 1 = very much worse, 2 = much worse, 3 = a little or minimally worse, 4 = no change, 5 = a little or minimally improved, 6 = much improved, and 7 = very much improved. Higher analytic scores therefore represented greater perceived improvement. The PGI-I and CGI-I were treated as brief patient- and clinician-centred global ratings of change between successive observed sessions (Busner & Targum, 2007; Mohebbi et al., 2018; Viktrup et al., 2012). Because both were single-item measures, internal consistency was not applicable.

### Study procedures

Recruitment began by inviting eligible therapists to participate in the study. Therapists who expressed interest identified one potentially eligible client with whom they were conducting ongoing individual psychotherapy or counselling. The therapists briefly introduced the study to an eligible client using a standardised recruitment script, after which the research team contacted interested clients directly. Participation was voluntary, and refusal to participate or withdrawal from the study did not affect the client’s access to treatment or the therapist’s professional relationship with the client.

All recruitment, informed-consent, and research-assessment procedures were conducted electronically through Google Forms. Therapists were first provided with a separate electronic informed-consent form describing the study’s purpose, procedures, expected time commitment, potential risks and benefits, confidentiality protections, voluntary nature of participation, and their right to decline or withdraw without penalty. After providing electronic consent, each therapist used a standardised recruitment script to inform one potentially eligible client about the study. Therapists did not obtain consent on behalf of their clients. Instead, clients who expressed interest were contacted directly by the research team and received a separate client-specific electronic informed-consent form containing the same relevant information and emphasising that participation or nonparticipation would not affect their access to treatment or their relationship with the therapist. Each participant indicated consent electronically before accessing any research questionnaires, and a dyad was enrolled only after both the client and therapist had independently provided informed consent.

After consent was obtained from both members, the dyad was assigned a nonidentifying study code used to link their responses across sessions. Following each observed session, clients and therapists received separate questionnaire links and completed parallel post-session measures independently. Neither participant could view the other dyad member’s responses, and individual ratings were not disclosed to the other member during the study.

Clients completed the WAI-SR client form, SES-*P* short form, PGI-S, and, beginning at the second observed session, PGI-I. Therapists completed the WAI-SR therapist form, SES-T short form, CGI-S, and, beginning at the second observed session, CGI-I. Measures were completed immediately after each session whenever possible to minimise recall bias and preserve the session-specific nature of the appraisals. Dyads were observed across eight to ten consecutive sessions. If a dyad missed a session assessment or terminated treatment before completing all observed sessions, available paired data were retained when analytically appropriate. Consistent with mixed-effects approaches to unbalanced longitudinal data, the primary analyses used all available observations under restricted maximum likelihood estimation, assuming data were missing at random conditional on observed variables (Twisk et al., 2013). Because the primary longitudinal APIM examined lagged associations from session t to session t + 1, dyads were included in the primary analysis if they provided at least four paired post-session assessments, yielding at least three adjacent lagged intervals. This threshold was specified a priori as a data-sufficiency criterion for estimating within-dyad session-to-session associations, given that L-APIM estimation depends on the number of dyads, repeated observations, and missing-data patterns (Lafit et al., 2022). Dyads with fewer paired observations were retained for descriptive analyses only.

### Data analysis

All analyses were conducted in R (R Core Team, 2025). Client and therapist data were matched using nonidentifying dyad codes and observed session numbers, then organised in long format, with each row representing one dyad-session observation. Data were screened for duplicate entries, unmatched responses, out-of-range values, missing session numbers, and inconsistent identifiers. Client and therapist WAI-SR mean scores were computed separately. Session quality was indexed using SES-*P* and SES-T short-form scores. PGI-S, PGI-I, CGI-S, and CGI-I were treated as single-item global ratings. Descriptive statistics, distributions, and missing-data patterns were examined before model estimation. Internal consistency reliability was estimated for multi-item measures; reliability was not computed for single-item global ratings. Missing data were handled using model-wise available-case estimation; therefore, each model used all dyad-session transitions with complete data for the variables included in that specific analysis.

Descriptive analyses summarised client and therapist ratings across sessions, including means, standard deviations, ranges, session trends, and client–therapist correspondence. Variance decomposition was used to estimate within-dyad and between-dyad variability, thereby assessing whether the variables showed sufficient session-to-session variation for longitudinal modelling. Therapeutic alliance ratings were decomposed into between-dyad and within-dyad components separately for clients and therapists. For each dyad member, the average alliance score across observed sessions represented the between-dyad component, while deviations from that person’s average represented session-specific within-dyad fluctuations. This centring strategy allowed the models to examine whether higher- or lower-than-usual alliance ratings predicted subsequent appraisals while accounting for stable differences in average alliance.

No multiple imputation was performed. Each lagged model used the dyad-session transitions with complete data for the outcome at t + 1, the corresponding prior outcome at t, both alliance predictors, the observed-session index, and the two between-dyad alliance means.

Longitudinal actor–partner interdependence models were estimated for distinguishable dyads. Separate models were fitted for session quality, clinical severity, and global improvement. For each outcome, client- and therapist-rated appraisals at session t + 1 were predicted from client- and therapist-rated alliance at session t, prior appraisal scores at session t, observed session number, and between-dyad alliance components. Actor effects referred to each dyad member’s alliance predicting their own subsequent appraisal, whereas partner effects referred to one dyad member’s alliance predicting the other member’s subsequent appraisal. Models included random intercepts for dyads to account for repeated observations nested within client–therapist pairs. Because each therapist contributed only one client, no therapist-level random effect was estimated. Session quality was treated as the primary outcome, whereas clinical severity and global improvement were examined as secondary outcomes. Global improvement models began after the first improvement rating because PGI-I and CGI-I required a preceding-session reference point.

Linear mixed-effects models were estimated using lme4 (Bates et al., 2015), with fixed-effect tests evaluated using Satterthwaite-approximated degrees of freedom implemented in lmerTest. Model diagnostics and performance indices were examined using performance (Lüdecke et al., 2021), and pairwise comparisons of domain-specific slopes were conducted with Tukey adjustment for multiple comparisons. The analytic approach followed recommendations for longitudinal dyadic data and intensive longitudinal APIM (Gistelinck & Loeys, 2019; Lafit et al., 2022). Models used complete data for all variables required for each lagged transition. Restricted maximum-likelihood estimation accommodated unbalanced numbers of available outcome observations, but observations missing required predictor values were not included. Simulation-based sensitivity analyses using the PowerLAPIM framework were conducted to estimate statistical power to detect prespecified standardised actor and partner effects of β = .20 with 54 dyads across eight to ten sessions (Lafit et al., 2022).

### Ethical considerations

The study was reviewed and approved by the University of the Philippines, College of Social Sciences and Philosophy Ethics Review Board (Approval No. CSSPERB-2023-091).

## Results

### Missing data and assessment completion

Missingness was present but expected with the intensive repeated-measures design. Across 471 observed session rows, 61 client forms were incomplete or missing (13.0%) and 42 therapist forms were incomplete or missing (8.9%); at least one form was incomplete in 83 rows. Recorded reasons included a missed or unreturned client form (n = 27), a partially completed client form (n = 14), a missed or unreturned therapist form (n = 19), a partially completed therapist form (n = 3), neither form being returned (n = 9), a technical or scheduling disruption (n = 7), and unavailable session documentation (n = 4). Some dyads also ended observation before the planned maximum number of sessions. The analyses therefore used model-specific complete adjacent-session transitions: 208 for session quality, 283 for severity, and 236 for improvement (see Table 1).

**Table 1.** Within- and between-dyad intercorrelations among alliance and appraisal variables.

Variable	1	2	3	4	5	6	7	8
1. Client WAI-SR	−	.53***	−.07	.07	.11	−.05	−.13	−.12
2. Therapist WAI-SR	.03	−	−.25	.00	.28*	−.01	−.07	−.18
3. Client session quality	.06	−.02	−	.07	.00	.20	.08	−.01
4. Therapist session quality	.04	.12*	.02	−	−.05	−.03	.14	−.19
5. Client severity	.05	.00	.04	−.01	−	.47***	−.22	−.25
6. Therapist severity	.04	.11*	−.02	.12*	.35***	−	−.17	−.27*
7. Client improvement	.07	−.03	.07	−.08	−.39***	−.43***	−	.28*
8. Therapist improvement	.03	−.02	.17**	−.08	−.38***	−.42***	.50***	−

*p < .05. **p < .01. ***p < .001.

Note: Coefficients below the diagonal are within-dyad correlations based on cluster-mean-centred session-level observations; pairwise session-level sample sizes ranged from 308 to 426. Coefficients above the diagonal are between-dyad correlations based on dyad-specific mean scores (*N* = 54 dyads). Positive improvement scores indicate greater perceived improvement. WAI-SR = Working Alliance Inventory–Short Revised. Correlations and significance tests were calculated using pairwise available observations.

### Descriptive statistics

Across observed sessions, client- and therapist-rated alliance had very similar mean levels (see Table 2). Client-rated WAI-SR scores averaged 3.53 (SD = 0.77), while therapist-rated WAI-SR scores averaged 3.53 (SD = 0.62). Session-quality ratings were moderate on average, with client SES-*P* ratings averaging 2.96 (SD = 1.13) and therapist SES-T ratings averaging 3.11 (SD = 1.02). Client-rated severity averaged 3.60 (SD = 1.57), whereas therapist-rated severity averaged 3.40 (SD = 1.51). Global improvement ratings were generally favourable, with client-rated improvement averaging 4.97 (SD = 1.02) and therapist-rated improvement averaging 5.13 (SD = 1.01).

**Table 2.** Descriptive statistics and intraclass correlations.

Measure	*n*	M	SD	Median	Range	ICC
Client WAI-SR	409	3.53	0.77	3.58	1.25–5.00	.83
Therapist WAI-SR	426	3.53	0.62	3.58	1.36–5.00	.76
Client session quality, SES-P	384	2.96	1.13	3.00	1.00–5.00	.12
Therapist session quality, SES-T	391	3.11	1.02	3.00	1.00–5.00	.16
Client severity, PGI-S	410	3.60	1.57	4.00	1.00–7.00	.70
Therapist severity, CGI-S	429	3.40	1.51	3.00	1.00–7.00	.58
Client improvement, PGI-I	358	4.97	1.02	5.00	2.00–7.00	.00
Therapist improvement, CGI-I	376	5.13	1.01	5.00	2.00–7.00	.00

Note: WAI-SR = Working Alliance Inventory–Short Revised; SES-*P* = Session Evaluation Scale–Patient Version; SES-T = Session Evaluation Scale–Therapist Version; PGI-S = Patient Global Impression–Severity; CGI-S = Clinical Global Impression–Severity; PGI-I = Patient Global Impression–Improvement; CGI-I = Clinical Global Impression–Improvement. ICC values represent dyad-level variance decomposition from random-intercept models.

Alliance ratings showed substantial between-dyad clustering, with ICCs of .83 for client-rated alliance and .76 for therapist-rated alliance. Severity ratings also showed substantial dyad-level clustering, with ICCs of .70 for client-rated severity and .58 for therapist-rated severity. In contrast, session-quality ratings showed relatively low dyad-level clustering, with ICCs of .12 for client session quality and .16 for therapist session quality, indicating more session-to-session variability. Global improvement ratings had ICCs near zero, indicating little detectable between-dyad variance relative to within-dyad and residual variance.

Table 1 presents within-dyad correlations below the diagonal and between-dyad correlations among person-specific means above the diagonal. The same-rater associations between alliance and session quality were small. Client-rated WAI-SR and SES-*P* were weakly associated within dyads, r = .06, *p* = .300, and were not associated between dyads, r = −.07, *p* = .615. Therapist-rated WAI-SR and SES-T showed a small within-dyad association, r = .12, *p* = .034, but essentially no between-dyad association, r < .01, *p* = .974. These estimates suggest that alliance and session-quality ratings were related but not redundant constructs.

#### Objective 1: descriptive longitudinal patterns

**Figure 1.**
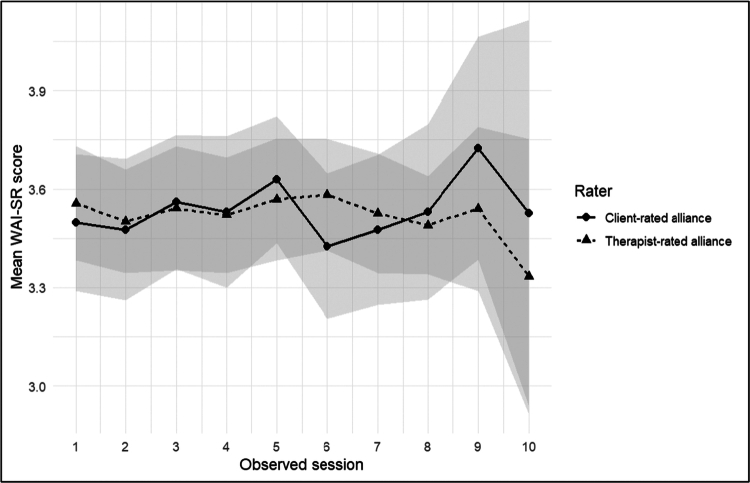
Client- and therapist-rated alliance trajectories across observed sessions. Note: Points represent mean Working Alliance Inventory–Short Revised (WAI-SR) scores at each observed session. Lines distinguish client-rated and therapist-rated alliance trajectories. Shaded grey bands represent approximate 95% confidence intervals around the session-level means. The number of contributing dyads declined across later sessions; accordingly, confidence intervals are wider at Sessions 9 and 10. Higher scores indicate stronger perceived therapeutic alliance.

**Figure 2.**
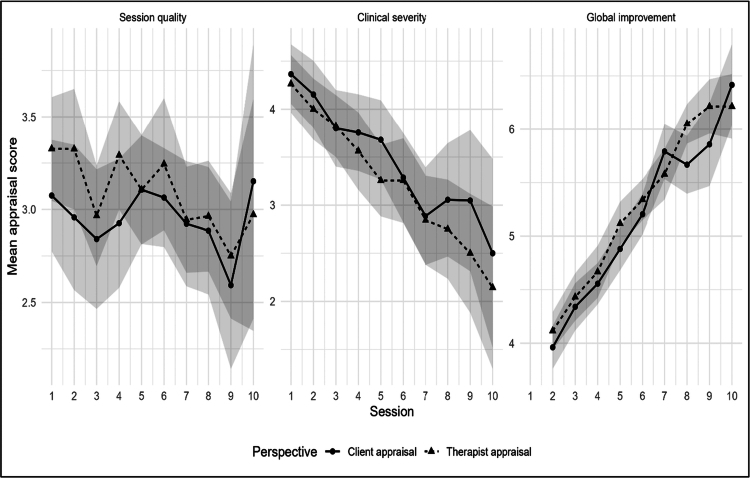
Client and therapist appraisal trajectories across observed sessions. Note: Points represent session-level mean raw appraisal scores, and shaded grey bands represent approximate 95% confidence intervals. The number of contributing dyads declined across later sessions; accordingly, confidence intervals are wider at Sessions 9 and 10. Session quality reflects client-rated SES-P and therapist-rated SES-T scores, clinical severity reflects client-rated PGI-S and therapist-rated CGI-S scores, and global improvement reflects client-rated PGI-I and therapist-rated CGI-I scores. Improvement ratings begin at Session 2 because global improvement ratings require comparison with a prior reference point. Because the three appraisal domains used different response scales, vertical levels should be interpreted within rather than across panels.

Figures 1 and 2 present the raw alliance and appraisal trajectories across observed sessions. Therapeutic alliance scores remained relatively stable across observed sessions, with client- and therapist-rated alliance generally clustering in the mid-to-high range of the WAI-SR scale. Session-quality ratings fluctuated more visibly across sessions, consistent with their lower ICCs and greater session-specific variability. Severity ratings declined across observed sessions for both clients and therapists. Client-rated severity decreased from M = 4.37 at Session 1 to M = 2.50 at Session 10, while therapist-rated severity decreased from M = 4.26 at Session 1 to M = 2.14 at Session 10. Global improvement ratings increased across sessions. Client-rated improvement rose from M = 3.96 at Session 2 to M = 6.42 at Session 10, while therapist-rated improvement increased from M = 4.12 at Session 2 to M = 6.21 at Session 10.

These descriptive patterns indicate that clinical severity and global improvement ratings showed the clearest time-ordered patterns of clinical change, whereas session quality varied more from session to session.

### Primary lagged actor–partner interdependence models

The primary L-APIM analyses examined whether client and therapist alliance ratings at session t predicted client and therapist appraisals at session t + 1. All models controlled for the corresponding prior appraisal at session t, observed session number, and between-dyad alliance means. Standardised estimates are reported to support comparison across outcome domains.

#### Model diagnostics and assumption checks

All six primary L-APIM models converged. The standardised models included random intercepts for dyad and fixed effects for prior outcome, observed session number, client and therapist alliance fluctuations, and between-dyad alliance means. Across the six primary models, maximum VIF values ranged from 1.37 to 1.66, indicating no problematic multicollinearity among predictors. However, all models were flagged as singular or near-singular, suggesting that random-intercept variance was negligible or difficult to estimate after accounting for the fixed effects. This means that baseline differences between dyads were very small or close to zero after the model had already accounted for the fixed predictors. Because the estimated dyad-level random-intercept variance was negligible, fixed-effect estimates were retained as the primary inferential results.

Marginal R^2^ values suggested that fixed effects explained approximately 25% of the variance in client session quality and 19% of the variance in therapist session quality. The severity models showed substantially larger marginal R^2^ values, .80 for client severity and .82 for therapist severity, largely reflecting strong autoregressive effects of prior severity. Global improvement models showed moderate levels of explained variance, with marginal R^2^ values of .35 for client-rated improvement and .38 for therapist-rated improvement.

#### Objective 2: actor effects

Table 3 shows the actor and partner effects. Both session-quality actor associations were statistically significant. For clients, higher-than-usual client-rated alliance at session t predicted higher client-rated session quality at session t + 1, *β* = .20, SE = .07, t(208) = 3.00, *p* = .003, 95% CI [.07, .33]. Similarly, for therapists, higher-than-usual therapist-rated alliance predicted higher therapist-rated session quality at the next session, *β* = .19, SE = .07, t(208) = 2.84, *p* = .005, 95% CI [.06, .32]. Thus, when clients or therapists experienced the alliance as stronger than usual in a given session, they tended to evaluate the following session more favourably. For clinical severity, the client actor effect was not significant, *β* = −.03, *p* = .322. However, the therapist actor effect was significant and negative, *β* = −.09, SE = .03, t(283) = −3.28, *p* = .001, 95% CI [−.14, −.03]. This indicates that when therapists rated the alliance as stronger than usual, they subsequently rated client severity as lower. Actor effects were not significant for either client- or therapist-rated global improvement.

**Table 3.** Standardised lagged actor and partner effects of alliance on subsequent appraisals.

Domain	Perspective	Effect	β	*SE*	*t*	*p*	95% CI
Session quality	Client	Actor	.20**	.07	3.00	.003	[.07, .33]
		Partner	.07	.06	1.14	.257	[−.05, .20]
	Therapist	Actor	.19**	.07	2.84	.005	[.06, .32]
		Partner	.06	.07	0.92	.360	[−.07, .19]
Clinical severity	Client	Actor	−.03	.03	−0.99	.322	[−.08, .03]
		Partner	−.02	.03	−0.63	.527	[−.07, .04]
	Therapist	Actor	−.09**	.03	−3.28	.001	[−.14, −.03]
		Partner	.00	.03	0.19	.853	[−.05, .06]
Global improvement	Client	Actor	.06	.05	1.07	.285	[−.05, .16]
		Partner	.05	.05	0.85	.394	[−.06, .15]
	Therapist	Actor	.06	.05	1.16	.248	[−.04, .16]
		Partner	.01	.05	0.26	.796	[−.09, .11]

Note: *β* values are standardised fixed-effect estimates. Actor effects refer to a dyad member’s own alliance fluctuation predicting that same member’s subsequent appraisal. Partner effects refer to one dyad member’s alliance fluctuation predicting the other dyad member’s subsequent appraisal. All models controlled for prior appraisal, observed session number, and between-dyad alliance means. *p < .05. **p < .01.

#### Objective 3: partner effects

No significant partner effects emerged in the primary models. Therapist alliance fluctuations did not significantly predict subsequent client-rated session quality, client-rated severity, or client-rated improvement. Likewise, client alliance fluctuations did not significantly predict subsequent therapist-rated session quality, therapist-rated severity, or therapist-rated improvement. These findings suggest that, in the present data, alliance fluctuations were more clearly associated with one’s own subsequent appraisals than with the other dyad member’s subsequent appraisals.

#### Objective 4: within-dyad alliance fluctuations

Because the focal alliance predictors were within-dyad centred, the actor and partner effects reflect session-specific deviations from each dyad member’s own average alliance rating. The findings therefore indicate that within-dyad alliance fluctuations had domain-specific implications. Client alliance fluctuations predicted subsequent client-rated session quality, therapist alliance fluctuations predicted subsequent therapist-rated session quality, and therapist alliance fluctuations predicted lower subsequent therapist-rated clinical severity. In contrast, within-dyad alliance fluctuations did not significantly predict global improvement ratings.

Prior appraisals were strong predictors of subsequent appraisals for both session quality and clinical severity. Client session quality at session t predicted client session quality at session t + 1, *β* = .41, *p* < .001, and therapist session quality at session t predicted therapist session quality at session t + 1, *β* = .35, *p* < .001. Clinical severity showed even stronger autoregressive stability, with client prior severity predicting subsequent client severity, *β* = .90, *p* < .001, and therapist prior severity predicting subsequent therapist severity, *β* = .92, *p* < .001.

For global improvement models, prior improvement ratings were not significant after accounting for observed session number and alliance variables. Observed session number was the strongest predictor of improvement, indicating that both client and therapist improvement ratings were higher at later points within the study observation window. Specifically, observed session number predicted client-rated improvement, *β* = .71, *p* < .001, and therapist-rated improvement, *β* = .66, *p* < .001.

Between-dyad alliance means were generally not associated with subsequent appraisals. Exceptions appeared in the client severity model: higher average client-rated alliance was associated with lower subsequent client severity, *β* = −.06, *p* = .045, whereas higher average therapist-rated alliance was associated with higher subsequent client severity, *β* = .08, *p* = .014. Because the primary focus of the study was on within-dyad lagged actor–partner processes, these between-dyad findings should be interpreted as exploratory.

#### Objective 5: cross-domain comparisons of actor and partner effects

To test whether actor and partner effects differed across appraisal domains, stacked cross-domain models were estimated separately for client and therapist perspectives (see Table 4). In the client-perspective model, prior appraisal strongly predicted subsequent appraisal, F(1, 727) = 341.61, *p* < .001. The overall actor-alliance effect was significant, F(1, 727) = 4.99, *p* = .026, whereas the overall partner-alliance effect was not, F(1, 727) = 1.73, *p* = .189. Specifically, the actor slope was significant for session quality, *β* = .18, SE = .06, 95% CI [.06, .29], but not for clinical severity, *β* = −.01, SE = .05, 95% CI [−.10, .09], *p* = .894, or global improvement, *β* = .04, SE = .05, 95% CI [−.07, .15], *p* = .468. Although the interaction did not reach statistical significance, the pattern of simple slopes suggested possible variation in client actor effects across appraisal domains. The domain-by-partner-alliance interaction was not significant, F(2, 727) = 0.22, *p* = .802.

**Table 4.** Cross-domain comparisons of actor and partner alliance effects.

Perspective	Effect	Domain	β	*SE*	*p*	95% CI
Client	Actor	Session quality	.18**	.06	.003	[.06, .29]
		Clinical severity	−.01	.05	.894	[−.10, .09]
		Global improvement	.04	.05	.468	[−.07, .15]
	Partner	Session quality	.06	.06	.261	[−.05, .17]
		Clinical severity	.01	.05	.765	[−.08, .11]
		Global improvement	.04	.06	.435	[−.07, .15]
Therapist	Actor	Session quality	.16**	.06	.004	[.05, .27]
		Clinical severity	−.05	.05	.278	[−.15, .04]
		Global improvement	.08	.05	.123	[−.02, .19]
	Partner	Session quality	.06	.06	.332	[−.06, .17]
		Clinical severity	.03	.05	.549	[−.07, .12]
		Global improvement	.00	.05	.946	[−.10, .11]

Note: Slopes are estimated standardised simple slopes from stacked cross-domain models. **p < .01.

In the therapist-perspective model, prior appraisal also strongly predicted subsequent appraisal, F(1, 727) = 384.58, *p* < .001. The overall actor-alliance effect was significant, F(1, 727) = 4.42, *p* = .036, while the overall partner-alliance effect was not, F(1, 727) = 0.94, *p* = .334. Specifically, the actor slope was significant for session quality, *β* = .16, SE = .06, 95% CI [.05, .27], but not for clinical severity, *β* = −.05, SE = .05, 95% CI [−.15, .04], *p* = .278, or global improvement, *β* = .08, SE = .05, 95% CI [−.02, .19], *p* = .123. The domain-by-actor-alliance interaction was significant, F(2, 727) = 4.47, *p* = .012, indicating that therapist actor effects differed across appraisal domains. The domain-by-partner-alliance interaction, however, was not significant, F(2, 727) = 0.23, *p* = .798.

**Figure 3.**
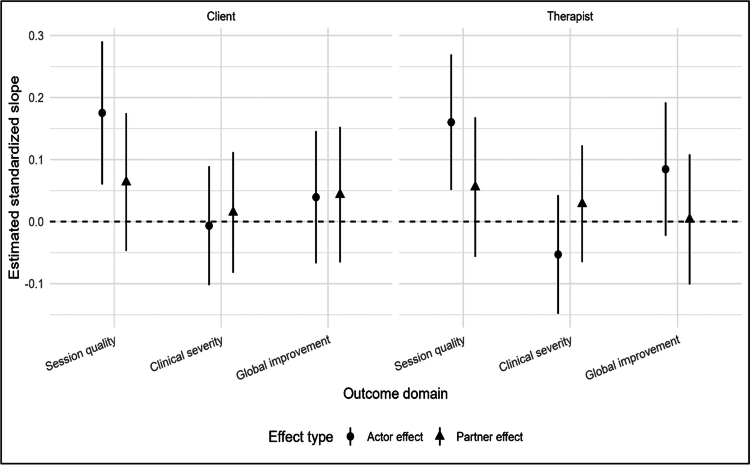
Cross-domain comparison of actor and partner alliance effects. Note: Points represent estimated standardised slopes; error bars represent 95% confidence intervals. Actor effects were most evident for session quality in both client and therapist perspectives, whereas partner effects were not statistically significant across appraisal domains.

As shown in Figure 3, actor effects were most evident for session quality. In the client model, the actor slope for session quality was significant, *β* = .18, 95% CI [.06, .29], while the actor slopes for clinical severity and global improvement were not significant. Pairwise comparisons showed that the client actor effect was significantly stronger for session quality than for clinical severity, estimate = .18, *p* = .046. In the therapist model, the actor slope for session quality was also significant, *β* = .16, 95% CI [.05, .27]. Pairwise comparisons showed that the therapist actor effect was significantly stronger for session quality than for clinical severity, estimate = .21, *p* = .011. Partner effects were not statistically significant and did not differ across domains.

#### Objective 6: actor–partner contrasts

Actor-minus-partner contrasts were estimated within each domain and perspective (see Table 5). Most actor–partner contrasts were not statistically significant. For session quality, actor effects were numerically larger than partner effects for both clients and therapists, but the contrasts did not reach significance. For client-rated severity and both improvement models, actor and partner effects did not differ significantly.

**Table 5.** Actor–partner contrasts within each outcome domain.

Domain	Perspective	Actor − Partner	SE	z	*p*	95% CI
Session quality	Client	.13	.10	1.31	.190	[−.06, .32]
	Therapist	.13	.10	1.26	.208	[−.07, .32]
Clinical severity	Client	−.01	.04	−0.24	.813	[−.08, .07]
	Therapist	−.09	.04	−2.40	.016	[−.16, −.02]
Global improvement	Client	.01	.08	0.13	.899	[−.14, .16]
	Therapist	.05	.07	0.63	.530	[−.10, .19]

Note: Positive values indicate larger actor than partner effects. Negative values indicate smaller or more negative actor effects relative to partner effects.

The only significant actor–partner contrast emerged for therapist-rated severity, estimate = −.09, SE = .04, z = −2.40, *p* = .016, 95% CI [−.16, −.02]. This indicates that, for therapist-rated severity, the therapist actor effect was significantly more negative than the partner effect. In substantive terms, therapists’ own alliance fluctuations were more strongly associated with their subsequent severity ratings than were clients’ alliance fluctuations.

### Power/sensitivity analysis

A simulation-based power analysis was conducted to evaluate the sensitivity of the lagged APIM session-quality models to detect moderate standardised actor and partner effects. Using 500 Monte Carlo simulations per effect, the models showed adequate power to detect a standardised effect of *β* = .20. Estimated power ranged from 87.2% to 88.4% across the four focal session-quality actor and partner effects. Specifically, power was 87.4% for the client actor effect on client-rated session quality, 87.2% for the therapist partner effect on client-rated session quality, 88.4% for the therapist actor effect on therapist-rated session quality, and 88.4% for the client partner effect on therapist-rated session quality. These results suggest that the design had adequate sensitivity to detect moderate within-dyad alliance effects in the session-quality models under the assumed data-generating model, missing-data structure, and random-effect specification.

### Summary of findings

Overall, the findings addressed the six research objectives. Descriptive trajectories showed that alliance ratings were relatively stable, whereas therapeutic appraisals changed in domain-specific ways: session quality fluctuated, clinical severity declined, and global improvement increased across observed sessions. Actor effects were most evident for session quality, with both clients’ and therapists’ stronger-than-usual alliance ratings predicting their own more favourable subsequent session-quality appraisals. A significant actor effect also emerged for therapist-rated severity, but actor effects were not consistently observed for global improvement. Partner effects were not statistically significant, indicating that one dyad member’s alliance fluctuation did not reliably predict the other member’s subsequent appraisal. Because alliance predictors were centred within dyads, these findings suggest that session-specific deviations from one’s typical alliance rating had selective predictive value, mainly for one’s own subsequent appraisals. Cross-domain analyses further showed that actor effects were strongest for session quality, while partner effects remained weak across domains. Actor effects were generally not significantly stronger than partner effects, except for therapist-rated severity, where the therapist actor effect was significantly more negative than the partner effect. Taken together, alliance fluctuations appeared most closely associated with one’s own subsequent session evaluation, whereas cross-person predictive effects were limited in this sample.

## Discussion

### Therapeutic alliance effects as proximal and perspective-dependent

The strongest and most consistent actor effects emerged for session quality. When clients rated the alliance as stronger than usual at a given session, they tended to rate the following session more positively. Similarly, when therapists rated the alliance as stronger than usual, they also tended to evaluate the following session more favourably. These results align with broader evidence that alliance is a robust predictor of psychotherapy process and outcome (Flückiger et al., 2018; Saxler et al., 2024). However, the present findings refine this general conclusion by showing that the alliance–appraisal association was clearest when the outcome was proximal to the session itself. Session quality is temporally and experientially close to alliance. Both are immediate post-session judgements, and both involve the client’s or therapist’s sense of whether the session was meaningful, collaborative, useful, or emotionally attuned. In this sense, the actor effects for session quality may reflect a continuity in each person’s own relational and evaluative frame: when the alliance feels stronger than usual, the next session is also more likely to be experienced as useful or effective.

Furthermore, the pattern of significant actor coefficients alongside nonsignificant partner coefficients suggests that alliance ratings may function partly as self-anchored appraisals. Although therapy is dyadic, clients and therapists do not necessarily experience or interpret the same relational event in identical ways. The finding that each person’s alliance rating predicted their own later session-quality appraisal, but not the other person’s later session-quality appraisal, supports prior APIM work showing that client and therapist perspectives can carry distinct implications for session evaluation and therapeutic process (Kivlighan et al., 2014; Markin et al., 2014; Zilcha-Mano et al., 2016). Rather than treating discrepancies between client and therapist alliance ratings as error or noise, these findings support the view that each perspective contains clinically meaningful information about that person’s experience of the therapeutic process.

### Therapeutic alliance actor effects as domain-specific

The cross-domain analyses showed that actor effects were strongest and most consistent for session quality. In contrast, alliance fluctuations were less consistently associated with clinical severity and global improvement. This domain-specific pattern is important because it cautions against assuming that alliance will have the same predictive meaning across all indicators of therapy process and progress. Session quality is an immediate experiential appraisal; clinical severity is a broader judgement of psychological difficulty; and global improvement requires a comparative evaluation of change over time. These domains differ in temporal scope, cognitive demands, and clinical meaning.

The relatively weaker alliance effects for global improvement may be explained by the cumulative nature of improvement ratings. Improvement is not simply a response to the most recent session or the immediately preceding alliance rating; it reflects an integrative judgement about change across the treatment period. In the present study, global improvement ratings increased strongly with observed session number, suggesting that perceived improvement was primarily tied to the passage of treatment time and cumulative therapeutic exposure. This is consistent with the idea that global improvement may be less sensitive to single-session alliance fluctuations than session quality. A client or therapist may perceive one session as relationally strong without immediately translating that experience into a global judgement that the client has improved.

The clinical severity models showed a different pattern. Prior severity was a strong predictor of subsequent severity, indicating high autoregressive stability. This means that clinical severity at one session strongly carried forward into the next session. Within this context, there may have been limited additional variance for alliance fluctuations to explain. The significant therapist actor effect for clinical severity is therefore notable: when therapists experienced the alliance as stronger than usual, they subsequently rated the client’s severity as lower. This may indicate that therapists’ own relational confidence or sense of collaborative engagement shaped their clinical judgement of the client’s functioning. Such a finding is consistent with evidence that therapist perspectives can uniquely relate to clinician-rated evaluations and treatment judgements (Constantino et al., 2020; Gaines et al., 2025; Li, 2022). At the same time, this finding should be interpreted cautiously because therapist-rated severity is a clinical appraisal rather than an objective indicator of symptom change.

### Limited partner effects and the meaning of dyadic influence

One of the most theoretically interesting findings was the absence of significant partner effects. Therapist alliance fluctuations did not significantly predict clients’ subsequent appraisals, and client alliance fluctuations did not significantly predict therapists’ subsequent appraisals. At first glance, this may appear inconsistent with the dyadic and relational nature of psychotherapy. However, the absence of partner effects does not mean that therapy is not interpersonal. Instead, it suggests that the measurable influence of one person’s alliance rating on the other person’s next appraisal may be more indirect, conditional, or delayed than can be captured through simple adjacent-session partner paths.

Several interpretations are possible. First, clients and therapists may respond to the relational process through their own internal appraisal systems. A therapist may sense that the alliance is strong, but the client may not experience that strength in the same way. Likewise, a client may feel understood and engaged, but the therapist may base clinical appraisals on a broader case formulation, observed functioning, risk, symptom persistence, or treatment goals. Second, partner effects may emerge more strongly during specific events such as ruptures, impasses, disclosure moments, or major shifts in treatment focus rather than across all sessions. Third, partner effects may operate through mediating processes such as emotional disclosure, task engagement, therapist responsiveness, or rupture repair rather than through direct lagged effects of alliance ratings. This interpretation is compatible with relationship-centred and evidence-based responsiveness models of psychotherapy, which conceptualise therapeutic change as interpersonal but not mechanically reciprocal (Norcross & Wampold, 2018). It also aligns with recent longitudinal psychotherapy research showing that alliance, expectations, symptom patterns, and interpersonal processes unfold dynamically across treatment (Iovoli et al., 2025; Li, 2022). The present results suggest that the dyadic nature of therapy may be better understood not as symmetrical cross-person prediction at every session, but as a more complex pattern of parallel, discrepant, and domain-specific appraisals.

### Filipino therapeutic contexts and culturally situated appraisal

The Filipino context of the study is important for interpreting the findings. The study situates alliance in relation to culturally mediated expectations regarding trust, authority, reciprocity, disclosure, and relational harmony. In such contexts, client and therapist appraisals may be shaped not only by what occurs in the session but also by how each person understands their role in the therapeutic relationship. Clients may hesitate to express dissatisfaction directly, particularly when therapists are perceived as authority figures or when maintaining relational harmony is valued. Therapists, in turn, may interpret politeness, compliance, or continued attendance as signs of engagement even when the client’s internal experience is more ambivalent. The limited partner effects may therefore reflect, in part, the partly private nature of each dyad member’s appraisal. A client’s alliance rating may not immediately alter the therapist’s subsequent appraisal if the client does not openly communicate dissatisfaction, uncertainty, or unmet expectations. Conversely, a therapist’s positive alliance rating may not translate into the client’s later appraisal if the client experiences the relationship through culturally shaped concerns about safety, deference, or whether it is acceptable to disagree. This interpretation is consistent with multicultural orientation scholarship, which emphasises that therapeutic processes are shaped by cultural humility, power, identity, and responsiveness (Davis et al., 2018; Orlowski et al., 2025). In Filipino psychotherapy, therapeutic alliance may be understood through *pakikibahagi ng loob*, or the active sharing of relational reality through which client and therapist gradually develop trust, mutual recognition, and relational safety (Rilveria, 2024). From this perspective, alliance is not a single, uniformly shared state but a relational process that may be experienced differently by each member of the dyad. This cultural framing is consistent with the present findings, in which client- and therapist-rated alliance primarily predicted each person’s own subsequent appraisals rather than those of the other dyad member. The observed pattern of actor effects is consistent with the possibility that clients and therapists may participate in the same therapeutic relationship while assigning different meanings to its quality, safety, usefulness, and clinical implications. Thus, examining their perspectives separately is especially important in a Filipino context, where deference, indirect communication, and the preservation of relational harmony may limit the immediate expression or recognition of disagreement. The absence of significant partner effects should therefore not be interpreted as evidence that the alliance is nonrelational; rather, it may indicate that *pakikibahagi ng loob* develops unevenly, remains partly private, or is not always translated into observable cross-person changes in the following session.

At the same time, caution is warranted. The present study did not directly measure cultural values, communication norms, deference, indirectness, or rupture markers. Therefore, cultural explanations should be framed as plausible contextual interpretations rather than definitive mechanisms. Future Filipino psychotherapy research would benefit from directly measuring culturally salient relational processes, such as comfort with disagreement, perceived therapist authority, relational safety, indirect communication, and culturally grounded expectations of care.

### Clinical and training implications

Several clinical implications follow from the findings. First, routine alliance monitoring should be bidirectional. Because client and therapist alliance ratings showed distinct actor effects, both perspectives matter. Client ratings are not merely satisfaction scores, and therapist ratings are not merely clinical impressions. Each provides information about how that person is experiencing the therapeutic relationship and how that experience may shape their subsequent session appraisal. Second, alliance monitoring should be used dialogically rather than evaluatively. The findings do not support using alliance scores simply as therapist performance metrics. Instead, alliance ratings should be treated as openings for collaborative enquiry: What felt helpful in the session? What felt unclear or misattuned? Did the client and therapist experience the same session differently? This is consistent with feedback-informed care and routine outcome monitoring approaches, which emphasise the clinical value of tracking process and progress over time (McAleavey et al., 2024). Third, therapists should be especially attentive to how their own alliance perceptions shape clinical judgement. The significant therapist actor effect for clinical severity suggests that when therapists feel more positively about the alliance, they may subsequently view the client as less severe. This may reflect valid clinical sensitivity: stronger collaboration may indeed accompany better functioning. However, it may also reflect therapist optimism, confirmatory bias, or overreliance on relational comfort as an indicator of clinical progress. Supervision can help therapists examine whether their sense of alliance is appropriately calibrated with client-reported experience, symptom data, and observed functioning. Fourth, the findings support alliance-focused supervision and therapist training. Trainees should learn to compare client and therapist ratings, notice discrepancies without defensiveness, and invite feedback in culturally responsive ways. In Filipino clinical contexts, this may require therapists to make disagreement explicitly safe, normalise client ambivalence, and attend to indirect signs of rupture or disengagement. Cultural humility and rupture repair should therefore be framed not as optional relational skills but as central clinical competencies (Boswell et al., 2025; Orlowski et al., 2025).

### Limitations and directions for future research

Several limitations should be considered. First, the study used a relatively modest sample of 54 dyads, although the repeated-measures design produced a larger number of session-level observations. The design was appropriate for intensive longitudinal dyadic modelling, but small or conditional partner effects may still have been difficult to detect. Future studies with larger dyadic samples could test whether partner effects emerge more clearly under specific conditions, such as early treatment, rupture episodes, severe symptom presentations, or high client–therapist discrepancy. Second, the study did not record the absolute course-of-treatment session number or the number of sessions completed before enrolment. The observed-session index therefore identifies each session's position within the study window. Consequently, the descriptive trajectories cannot establish how alliance or appraisal changed from treatment onset, and the analyses could not test whether actor, partner, or convergence patterns differed according to relationship duration. The lagged models controlled prior appraisal and therefore provide stronger temporal ordering than concurrent associations, but they do not eliminate reciprocal causation: favourable prior change or a positively experienced session may also shape subsequent alliance ratings. Future studies should enrol dyads at treatment onset or record cumulative session count and use cross-lagged, dynamic structural equation, or other reciprocal longitudinal models to distinguish alliance-to-appraisal and appraisal-to-alliance pathways (Zilcha-Mano & Fisher, 2022). Third, the study relied on brief post-session self-report and clinician-report measures. Although these measures are clinically efficient and appropriate for repeated session-level assessment, single-item clinical severity and global improvement ratings may not capture the full complexity of psychological distress, functioning, or treatment response. Future studies should include multi-item symptom measures, functional outcomes, dropout, rupture markers, and qualitative accounts of client and therapist experience. Fourth, missing data were handled using model-wise available-case estimation. This approach allowed each model to use all complete transitions available for that analysis, but it assumes that missingness was sufficiently accounted for by observed variables. Future work may compare available-case estimates with multiple imputation, full-information approaches, or sensitivity analyses that model dropout and missed session assessments more explicitly. Fifth, all models were flagged as singular or near-singular, indicating that random-intercept variance was limited after accounting for fixed predictors. Singularity does not necessarily render the fixed-effect estimates unusable, but it warrants caution regarding their uncertainty and the adequacy of the random-effects specification. Future studies may benefit from larger samples, more measurement occasions, or models that include random slopes when the data structure permits. Sixth, the cultural interpretation of the findings remains indirect. Although the study was conducted with Filipino client–therapist dyads, it did not directly test cultural mechanisms such as deference, relational harmony, indirect communication, or culturally shaped expectations of therapist authority. Future research should incorporate culturally grounded measures and qualitative interviews to clarify how Filipino clients and therapists construct meaning around alliance, disagreement, improvement, and clinical severity. Finally, the study examined adjacent-session lagged effects. Alliance processes may operate across different temporal windows. Some alliance experiences may shape immediate session quality, whereas others may influence improvement only after several sessions. Future studies could test nonlinear trajectories, delayed effects, rupture-repair sequences, and dynamic systems models of client–therapist interaction.

## Conclusion

The present study contributes to psychotherapy and counselling research by showing that therapeutic alliance is a dyadic but perspective-specific process whose implications vary across appraisal domains. Alliance fluctuations were most predictive of each dyad member’s own subsequent session-quality appraisal, while partner effects were limited. Therapist-rated alliance also predicted lower subsequent therapist-rated clinical severity, suggesting that therapists’ relational experience may shape their clinical judgements. Cross-domain analyses further showed that alliance effects were strongest for session quality, underscoring the proximal link between alliance and how sessions are experienced. These findings support a nuanced view of alliance as more than a general predictor of outcome. Alliance appears to function as a session-level relational signal that is interpreted through each dyad member’s own perspective, role, and evaluative frame. Clinically, the findings underscore the importance of bidirectional alliance monitoring, culturally responsive feedback practices, and supervision that helps therapists examine both client and therapist perspectives. Rather than asking only whether the alliance is strong, clinicians and researchers should continue asking whose alliance perception is being considered, whose appraisal it shapes, and how these meanings unfold across the course of therapy.

## Supplementary Material

Supplementary Material Supplementary Material.docx

## Data Availability

Deidentified data and materials supporting the findings of this study are available from the corresponding author upon reasonable request, subject to ethical and confidentiality restrictions.
