# Supplementary material for: Whose alliance shapes whose appraisal? Longitudinal actor–partner associations between therapeutic alliance and session quality, clinical severity, and global improvement among Filipino client–therapist dyads
Source: Cogent Ment Health. 2026 Aug 11;5(1):2716621. doi: 10.1080/28324765.2026.2716621 (PMC13463515; doi:10.1080/28324765.2026.2716621)
Supplement: Supplementary file 1 [file OAMH_A_2716621_SM1658.docx]

**Supplementary Material**

**Study Instruments**

*Whose Alliance Shapes Whose Appraisal? Longitudinal Actor–Partner Associations Between Therapeutic Alliance and Session Quality, Clinical Severity, and Global Improvement Among Filipino Client–Therapist Dyads*

**Contents.** This supplementary file presents the client and therapist forms of the Working Alliance Inventory–Short Revised, the Session Evaluation Scale, and the global impression measures of severity and improvement used in the study.

Appendix S1. Working Alliance Inventory–Short Revised: Client Version

Appendix S2. Working Alliance Inventory–Short Revised: Therapist Version

Appendix S3. Session Evaluation Scale–Patient Form

Appendix S4. Session Evaluation Scale–Therapist Form

Appendix S5. Clinical Global Impression–Severity

Appendix S6. Patient Global Impression–Severity

Appendix S7. Clinical Global Impression–Improvement

Appendix S8. Patient Global Impression–Improvement

**Appendix S1**

**Working Alliance Inventory–Short Revised (WAI-SR): Client Version**

**Instructions.** Below is a list of statements and questions about experiences people might have with their therapy or therapist. Some items refer directly to your therapist with a blank space. As you read the sentences, mentally insert the name of your therapist in place of _____ in the text. Think about your experience in therapy and decide which response category best describes your own experience.

**Important.** Please take your time to consider each question carefully.

**1.** As a result of these sessions, I am clearer as to how I might be able to change.

| 1 | 2 | 3 | 4 | 5 |
| --- | --- | --- | --- | --- |
| Seldom | Sometimes | Fairly Often | Very Often | Always |

**2.** What I am doing in therapy gives me new ways of looking at my problem.

| 1 | 2 | 3 | 4 | 5 |
| --- | --- | --- | --- | --- |
| Seldom | Sometimes | Fairly Often | Very Often | Always |

**3.** I believe _____ likes me.

| 1 | 2 | 3 | 4 | 5 |
| --- | --- | --- | --- | --- |
| Seldom | Sometimes | Fairly Often | Very Often | Always |

**4.** _____ and I collaborate on setting goals for my therapy.

| 1 | 2 | 3 | 4 | 5 |
| --- | --- | --- | --- | --- |
| Seldom | Sometimes | Fairly Often | Very Often | Always |

**5.** _____ and I respect each other.

| 1 | 2 | 3 | 4 | 5 |
| --- | --- | --- | --- | --- |
| Seldom | Sometimes | Fairly Often | Very Often | Always |

**6.** _____ and I are working toward mutually agreed-upon goals.

| 1 | 2 | 3 | 4 | 5 |
| --- | --- | --- | --- | --- |
| Seldom | Sometimes | Fairly Often | Very Often | Always |

**7.** I feel that _____ appreciates me.

| 1 | 2 | 3 | 4 | 5 |
| --- | --- | --- | --- | --- |
| Seldom | Sometimes | Fairly Often | Very Often | Always |

**8.** _____ and I agree on what is important for me to work on.

| 1 | 2 | 3 | 4 | 5 |
| --- | --- | --- | --- | --- |
| Seldom | Sometimes | Fairly Often | Very Often | Always |

**9.** I feel _____ cares about me even when I do things that he/she does not approve of.

| 1 | 2 | 3 | 4 | 5 |
| --- | --- | --- | --- | --- |
| Seldom | Sometimes | Fairly Often | Very Often | Always |

**10.** I feel that the things I do in therapy will help me accomplish the changes that I want.

| 1 | 2 | 3 | 4 | 5 |
| --- | --- | --- | --- | --- |
| Seldom | Sometimes | Fairly Often | Very Often | Always |

**11.** _____ and I have established a good understanding of the kind of changes that would be good for me.

| 1 | 2 | 3 | 4 | 5 |
| --- | --- | --- | --- | --- |
| Seldom | Sometimes | Fairly Often | Very Often | Always |

**12.** I believe the way we are working with my problem is correct.

| 1 | 2 | 3 | 4 | 5 |
| --- | --- | --- | --- | --- |
| Seldom | Sometimes | Fairly Often | Very Often | Always |

*Note.* Items copyright © Adam Horvath. Goal items: 4, 6, 8, and 11; task items: 1, 2, 10, and 12; bond items: 3, 5, 7, and 9.

**Appendix S2**

**Working Alliance Inventory–Short Revised: Therapist Version (WAI-SR-T)**

**Instructions.** Below are sentences that describe some of the different ways a person might think or feel about his or her client. As you read the sentences, mentally insert the name of your client in place of _____ in the text. If a statement describes the way you always feel or think, select 5; if it seldom applies to you, select 1. Use the numbers in between to describe variations between these extremes.

**1.** As a result of these sessions, _____ is clearer as to how he/she might be able to change.

| 1 | 2 | 3 | 4 | 5 |
| --- | --- | --- | --- | --- |
| Seldom | Sometimes | Fairly Often | Very Often | Always |

**2.** _____ and I both feel confident about the usefulness of our current activity in therapy.

| 1 | 2 | 3 | 4 | 5 |
| --- | --- | --- | --- | --- |
| Seldom | Sometimes | Fairly Often | Very Often | Always |

**3.** I believe _____ likes me.

| 1 | 2 | 3 | 4 | 5 |
| --- | --- | --- | --- | --- |
| Seldom | Sometimes | Fairly Often | Very Often | Always |

**4.** _____ and I have collaborated on setting goals for these sessions.

| 1 | 2 | 3 | 4 | 5 |
| --- | --- | --- | --- | --- |
| Seldom | Sometimes | Fairly Often | Very Often | Always |

**5.** _____ and I respect each other.

| 1 | 2 | 3 | 4 | 5 |
| --- | --- | --- | --- | --- |
| Seldom | Sometimes | Fairly Often | Very Often | Always |

**6.** We are working toward mutually agreed-upon goals.

| 1 | 2 | 3 | 4 | 5 |
| --- | --- | --- | --- | --- |
| Seldom | Sometimes | Fairly Often | Very Often | Always |

**7.** I appreciate _____ as a person.

| 1 | 2 | 3 | 4 | 5 |
| --- | --- | --- | --- | --- |
| Seldom | Sometimes | Fairly Often | Very Often | Always |

**8.** We agree on what is important for _____ to work on.

| 1 | 2 | 3 | 4 | 5 |
| --- | --- | --- | --- | --- |
| Seldom | Sometimes | Fairly Often | Very Often | Always |

**9.** I respect _____ even when he/she does things I do not approve of.

| 1 | 2 | 3 | 4 | 5 |
| --- | --- | --- | --- | --- |
| Seldom | Sometimes | Fairly Often | Very Often | Always |

**10.** I feel confident that the things we do in therapy will help _____ accomplish the changes that he/she desires.

| 1 | 2 | 3 | 4 | 5 |
| --- | --- | --- | --- | --- |
| Seldom | Sometimes | Fairly Often | Very Often | Always |

**11.** We have established a good understanding of the kind of changes that would be good for _____.

| 1 | 2 | 3 | 4 | 5 |
| --- | --- | --- | --- | --- |
| Seldom | Sometimes | Fairly Often | Very Often | Always |

**12.** _____ believes that the way we are working with his/her problems is correct.

| 1 | 2 | 3 | 4 | 5 |
| --- | --- | --- | --- | --- |
| Seldom | Sometimes | Fairly Often | Very Often | Always |

**Appendix S3**

**Session Evaluation Scale–Patient Form (SES-P)**

**Instructions.** Indicate how much each statement reflects your experiences in your most recent helping session. Please note that not all of these things occur in every session because helpers do many different things to be helpful.

**1.** I am glad I attended this session.

| 1 | 2 | 3 | 4 | 5 |
| --- | --- | --- | --- | --- |
| Strongly Disagree | Disagree | Neutral | Agree | Strongly Agree |

**2.** I did not feel satisfied with what I got out of this session.

| 1 | 2 | 3 | 4 | 5 |
| --- | --- | --- | --- | --- |
| Strongly Disagree | Disagree | Neutral | Agree | Strongly Agree |

**3.** I thought that this session was helpful.

| 1 | 2 | 3 | 4 | 5 |
| --- | --- | --- | --- | --- |
| Strongly Disagree | Disagree | Neutral | Agree | Strongly Agree |

**4.** I did not think that this session was valuable.

| 1 | 2 | 3 | 4 | 5 |
| --- | --- | --- | --- | --- |
| Strongly Disagree | Disagree | Neutral | Agree | Strongly Agree |

**5.** Rate the overall effectiveness of this session.

| 1 | 2 | 3 | 4 | 5 |
| --- | --- | --- | --- | --- |
| Not Effective |  |  |  | Effective |

*Scoring note.* Items 2 and 4 are reverse-scored.

*Ultra-short SES-P used in the present study.* Items 3, 4, and 5.

**Appendix S4**

**Session Evaluation Scale–Therapist Form (SES-T)**

**Instructions.** Indicate how much each statement reflects your experiences in your most recent helping session.

**1.** I am glad my patient attended this session.

| 1 | 2 | 3 | 4 | 5 |
| --- | --- | --- | --- | --- |
| Strongly Disagree | Disagree | Neutral | Agree | Strongly Agree |

**2.** I did not feel satisfied with what my patient got out of this session.

| 1 | 2 | 3 | 4 | 5 |
| --- | --- | --- | --- | --- |
| Strongly Disagree | Disagree | Neutral | Agree | Strongly Agree |

**3.** I thought that this session was helpful for my patient.

| 1 | 2 | 3 | 4 | 5 |
| --- | --- | --- | --- | --- |
| Strongly Disagree | Disagree | Neutral | Agree | Strongly Agree |

**4.** I did not think that this session was valuable for my patient.

| 1 | 2 | 3 | 4 | 5 |
| --- | --- | --- | --- | --- |
| Strongly Disagree | Disagree | Neutral | Agree | Strongly Agree |

**5.** Rate the overall effectiveness of this session for my patient.

| 1 | 2 | 3 | 4 | 5 |
| --- | --- | --- | --- | --- |
| Not Effective |  |  |  | Effective |

*Scoring note.* Items 2 and 4 are reverse-scored.

*Ultra-short SES-T used in the present study.* Items 3, 4, and 5.

**Appendix S5**

**Clinical Global Impression–Severity (CGI-S)**

**Item prompt.** Considering your total clinical experience with this particular population, how mentally ill is the patient at this time?

| **Score** | **Response anchor** |
| --- | --- |
| 0 | Not assessed |
| 1 | Normal, not at all ill |
| 2 | Borderline mentally ill |
| 3 | Mildly ill |
| 4 | Moderately ill |
| 5 | Markedly ill |
| 6 | Severely ill |
| 7 | Among the most extremely ill patients |

*Note.* Higher scores indicate greater clinician-rated severity. The severity score was not reverse-coded for analysis.

**Appendix S6**

**Patient Global Impression–Severity (PGI-S)**

**Item prompt.** Considering your overall psychological or emotional condition, how severe are your difficulties at this time?

| **Score** | **Response anchor** |
| --- | --- |
| 1 | None; not at all severe |
| 2 | Minimal |
| 3 | Mild |
| 4 | Moderate |
| 5 | Marked |
| 6 | Severe |
| 7 | Among the most extremely severe |

*Note.* The response continuum was aligned with the 1–7 clinician-rated severity scale. Higher scores indicate greater patient-rated severity. The severity score was not reverse-coded for analysis.

**Appendix S7**

**Clinical Global Impression–Improvement (CGI-I)**

**Item prompt.** Rate total improvement, whether or not, in your judgment, it is due entirely to treatment. Compared with the client’s condition at the previous session, how much has the client improved?

| **Score** | **Response anchor** |
| --- | --- |
| 0 | Not assessed |
| 1 | Very much improved |
| 2 | Much improved |
| 3 | Minimally improved |
| 4 | No change |
| 5 | Minimally worse |
| 6 | Much worse |
| 7 | Very much worse |

*Note.* The scale was administered in its original direction, in which lower scores indicate greater improvement. For analysis, ratings from 1 to 7 were reverse-coded as 8 − original score so that higher analytic scores represented greater improvement.

**Appendix S8**

**Patient Global Impression–Improvement (PGI-I)**

**Item prompt.** Compared with the previous session, how would you describe how much you have improved?

| **Score** | **Response anchor** |
| --- | --- |
| 1 | Very much improved |
| 2 | Much improved |
| 3 | A little improved |
| 4 | No change |
| 5 | A little worse |
| 6 | Much worse |
| 7 | Very much worse |

*Note.* The scale was administered in its original direction, in which lower scores indicate greater improvement. For analysis, ratings were reverse-coded as 8 − original score so that higher analytic scores represented greater improvement.
